# Immunotherapy through the Lens of Non-Small Cell Lung Cancer

**DOI:** 10.3390/cancers15112996

**Published:** 2023-05-30

**Authors:** Robyn Stanley, Saoirse Flanagan, David O’ Reilly, Ella Kearney, Jarushka Naidoo, Catríona M. Dowling

**Affiliations:** 1 School of Medicine, University of Limerick, V94 T9PX Limerick, Ireland; 2 Beaumont Hospital, D09 V2N0 Dublin, Ireland; davidoreilly@rcsi.ie; 3 Department of Medicine, Royal College of Surgeons in Ireland, D02 YN77 Dublin, Ireland; 4 Sidney Kimmel Comprehensive Cancer Centre, Johns Hopkins University, Baltimore, MD 21218, USA

**Keywords:** non-small cell lung cancer, clinical trials, immune checkpoint inhibitors, immune-related adverse events, biomarkers

## Abstract

**Simple Summary:**

The landscape of non-small cell lung cancer has changed dramatically over the past decade. This is largely due to the introduction of immunotherapy, and in particular, immune checkpoint blockade inhibitors. Anti-PD-1 immunotherapy is now standard treatment for patients with non-small cell lung cancer. However, not all patients respond to immunotherapy, and few patients achieve long-term survival. Moreover, some patients experience adverse effects from the treatment. In this review, we explain the modes of actions of common immunotherapy strategies, summarise the clinical trials that have led to the widespread use of immunotherapy and present some current challenges in the field of immunotherapy.

**Abstract:**

Immunotherapy has revolutionised anti-cancer treatment in solid organ malignancies. Specifically, the discovery of CTLA-4 followed by PD-1 in the early 2000s led to the practice-changing clinical development of immune checkpoint inhibitors (ICI). Patients with lung cancer, including both small cell (SCLC) and non-small cell lung cancer (NSCLC), benefit from the most commonly used form of immunotherapy in immune checkpoint inhibitors (ICI), resulting in increased survival and quality of life. In NSCLC, the benefit of ICIs has now extended from advanced NSCLC to earlier stages of disease, resulting in durable benefits and the even the emergence of the word ‘cure’ in long term responders. However, not all patients respond to immunotherapy, and few patients achieve long-term survival. Patients may also develop immune-related toxicity, a small percentage of which is associated with significant mortality and morbidity. This review article highlights the various types of immunotherapeutic strategies, their modes of action, and the practice-changing clinical trials that have led to the widespread use of immunotherapy, with a focus on ICIs in NSCLC and the current challenges associated with advancing the field of immunotherapy.

## 1. Modes of Action for Immunotherapies 

### 1.1. Immune Checkpoint Blockade Inhibitors (ICIs)

The development of immune checkpoint inhibitors (ICIs) has earned much interest in the field of immuno-oncology due to their significant success, in particular with improved survival in patients with difficult-to-treat cancers such as NSCLC [1].

A key function of the immune system is to distinguish self from nonself. This is achieved by the detection and binding of a T cell receptor (TCR) to an antigen displayed by the major histocompatibility complex (MHC) on the surface of an antigen-presenting cell (APC) [2]. T cell activation is regulated by several immune checkpoint pathways during the immune response, a process called peripheral tolerance [2]. At the centre of this process are the cytotoxic T-lymphocytes-associated antigen 4 (CTLA-4) and programmed death 1 (PD-1) immune checkpoint pathways. Cancer cells have the ability to avoid immune suppression by expressing checkpoint molecules such as PD-1, CTLA4 and programmed death ligand 1 (PD-L1) [3]. 

**Figure 1:**
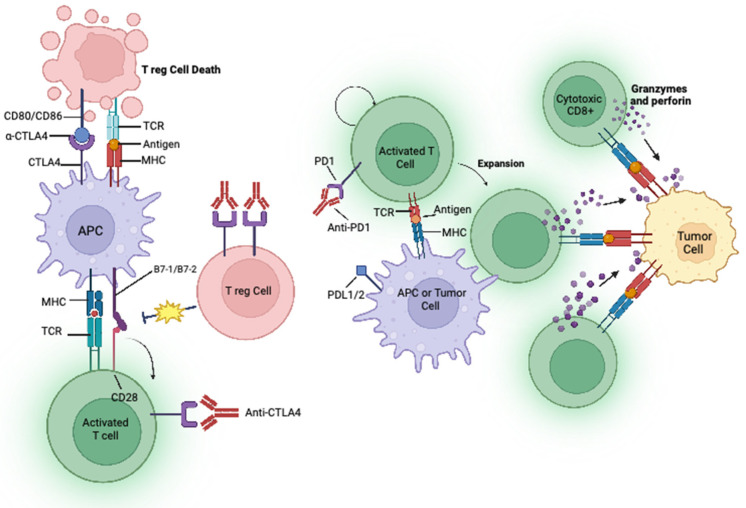
Mechanisms of action of immune checkpoint inhibitors. T cells recognize tumour antigens at the MHC on antigen presenting cells (APC) by T cell receptors (TCR) displayed on T cells. The interaction between CD80/CD86 (also known as B7-1 or B7-2) on APC and CD28 mediates a T cell co-stimulation in conjugation with TCR signals emitted by the T cell. CTLA-4 on activated T cells interacts with either CD80 or CD86 ligands (with a higher affinity for CD28), thus stopping the T cell from sending inhibitory signal to a T cells. Monoclonal antibodies, anti-CTLA-4 (e.g., ipilimumab) block this inhibitory pathway, thereby restoring T cell activity and amplifying an immune response. Cytotoxic CD8^+^ specific T cells recognise tumour antigens at the MHC of the APC using TCR. PD-1 is expressed on activated T cells, and the binding of PD-L1 on APC to PD-1 results in an adaptive expression. The interaction between PD-1 and PD-L1 negatively regulates the anti-tumour T cell response and causes immunosuppression. Anti-PD-1 (e.g., pembrolizumab) and anti-PD-L1 mAbs (e.g., atezoliumab) block this inhibitory pathway, thereby restoring T cell activity and relieving the immunosuppression.

PD-1 is a member of the CD28 family of negative costimulatory receptors expressed on activated lymphocytes and monocytes. It modifies T cell activation by binding to its ligands presented on APCs, i.e., PD-L1 and programmed death 2 (PD-L2). PD-1 plays an immunoregulatory role by reducing initial T cell activation, modifying T cell differentiation and effector functions, and supporting the development of immunological memory [2]. PD-L1 is expressed on tumour cells, and when it binds to PD-1 on the T cell, T cell-associated kinases are inhibited, preventing the development of cytotoxic T cell response to tumours [4], thus stopping T cells from identifying and eradicating tumour cells (Figure 1). Moreover, binding to PD-L1 can inhibit the proliferation of T lymphocytes and the production of cytokines such as IL-2 and IFN-Y, thus inhibiting the proliferation of B lymphocytes [4]. This results in an overall weakening of the immune response. High expression of PD-L1 is observed in 24–60% of patients with non-small cell lung cancer (NSCLC) [5], and this high expression of PD-L1 has been shown to result in a shorter survival and a poor prognosis for patients [4]. The expression of PD-L1 is controlled by a number of factors such as transcriptional regulation [6,7,8], epigenetic regulation [9,10], post-translational modifications [11,12,13] and metabolic reprogramming [14,15,16]. Monoclonal antibodies which bind and block PD-1 receptors signals can reactivate the tumour-infiltering lymphocytes, allowing the recognition and elimination of malignant cells [17]. 

CTLA-4 is a receptor on T cells that causes the inhibition of T cell priming, activation, and migration [18]. CTLA-4 is highly expressed on activated T cells where it competes with CD28 for binding to CD80 and CD86 expressed on APC (Figure 1) [19]. Consequently, the overexpression of CTLA-4 in the tumour micro-environment can act as a biomarker for prognosis and treatment of NSCLC [20]. Targeting CTLA-4 can prevent the immunosuppressive CTLA-4 binding mechanisms by diminishing the co-stimulatory binding of CD28. This allows for the activation and proliferation of T cells at early stages prior to complementary ligand binding, leading to stimulatory signals which attack cancer cells [2].

Currently six mAb targeting PD-1/PD-L1 and one mAb targeting CTLA4 have been approved by the FDA for the treatment of NSCLC (Table 1), which we will discuss in more depth in subsequent sections. 

There are also several other negative checkpoints emerging that suppress the immune system through ligand/receptor binding. These include T cell immunoglobulin, mucin-containing protein-3 (TIM-3), V-domain immunoglobulin suppressor of T cell activation (VISTA), ITIM domain T cell immunoreceptor (TIGIT), and Lymphocyte activation gene-3 (LAG-3) [30]. TIM-3 is expressed on activated CD4^+^ T cells (T helper cells) and negatively induces cytokines (Th1 and Th17). It also attaches to galectin-9, which leads to apoptosis of cytotoxic cells (CD4^+^ and CD8^+^) [31]. Galectin-9 is upregulated in cancer cells and suppresses an anti-tumour response through ligation with TIM-3 [31]. LAG-3 can also bind to galactin 3 in the TME, resulting in a reduction in anti-tumour response by inhibiting CD8^+^ T cells [32]. TIGIT inhibits the immune system by competing with CD226 to interact with CD112 and CD155 which would normally activate T cells [33]. VISTA is a member of the B7 family of checkpoints that are primarily expressed in hematopoietic cells and naïve CD4^+^ and Foxp3^+^ regulatory T cells. It can act as both a ligand and a receptor in negatively regulating immune responses [34]. VISTA has been shown to be more complicated than originally thought and its mechanism of action is not fully understood. 

Some new studies have suggested a dual combination of ICIs may prevent resistance and improve positive outcomes for patients; however, elevated side effects must be considered [30]. Patients who do respond to ICIs show that tumour load, immunogenicity, and the extent of immunosuppression in the microenvironment are critical factors that determine the probability of a positive clinical response [35]. Thus, ICI combination strategies could be the way forward. Dual regimes including anti-PD-1 in combination with less successful ICI such as anti-TIGIT and anti-LAG-3 are currently in clinical trials [35]. For example in the CITYSCAPE trial, atezolizumab (anti-PD-L1) plus tiragolumab (anti-TIGIT) are being assessed for first-line treatment of NSCLC [36]. Findings from this study have demonstrated that titragolumab plus atezolizumab improved both the response rate and progression free survival compared with atezolizumab alone with a safe profile [36]. Other combination strategies involving ICIs and other forms of immunotherapy include the use of adoptive cell transfer with ICIs. The most recent of which includes the use of nivolumab in combination with tumour infiltrating lymphocytes (TIL). A phase 1 clinical trial investigating the benefit of TILs administrated with nivolumab in patients with advanced NSCLC demonstrated effective T cell expansion in vivo and manageable toxicity to the patient [37], showing promise for the future. 

### 1.2. CAR-T Cell Therapy 

Another type of immunotherapy which has gained some attention in the lung cancer field is chimeric antigen receptor (CAR) T cells. CAR-T cells are T cells that have been genetically-modified to express the CAR protein, allowing T cells to recognize cancer cells, without relying on MHC [38], and trigger a downstream signalling cascade of T cell activation [39]. CAR-T cell therapy is an approved therapy for haematologic malignancies such as B-cell leukaemia [40]. Many studies have displayed successful anti-tumour activity in vitro [41] and in vivo [42] using CAR-T cells specific to NSCLC tumour-associated antigens such as B7-H3 [41], GD2 [42] and PTK7 [43]. These studies and others have led to the development of clinical trials of CAR-T cell therapy in NSCLC (Table 2). However, these trials are still in early phase and are yet to receive FDA approval. Current issues in using CAR-T cells to treat solid tumours include the harsh tumour microenvironment [44], resulting in T cell exhaustion and subsequent failure to activate their cytotoxic abilities. T cells are also unable to detect intracellular antigens [44], and most significantly, there has yet to be a surface antigen discovered that is as widely expressed as CD19 on B-cells seen in leukaemia [45].

### 1.3. Oncolytic Virus Therapy 

Oncolytic viruses are genetically modified to promote the targeting and destruction of specific cancer cells, while leaving self-cells untouched [46]. The genetic modifications used in oncolytic virus therapy (OVT) remove the adenoviral genes that are used in normal cells for viral infection, but not in cancer cells [47]. The primary mechanisms of oncolytic viruses include the lysis of tumour cells, followed by the promotion of anti-tumour activity [48], and changes in cytokine levels which generate a proinflammatory tumour microenvironment [49]. Studies have also shown the ability of OVT to promote expression of PD-L1 on tumour cells, making them better targets for ICIs [50].

OVT was first approved by the FDA as a treatment for unresectable melanoma patients in 2015 [51], and there are ongoing trials examining the use of OVT in NSCLC patients (Table 3). A phase I study demonstrated the successful insertion of these oncolytic viruses into NSCLC cells while avoiding healthy tissue, and no adverse reactions were reported [49]. The study assessed intravenous delivery (IV) of enadenotucirev (ColoAd1) in patients with resectable colorectal cancer, non-small cell lung cancer, urothelial cell cancer and renal cell cancer patients. Immunohistochemistry (IHC) analysis was utilised to investigate the success of IV delivery of the virus, and successful viral replication was observed, demonstrating effective delivery of the virus. Positive CD8^+^ T cell infiltration was also observed in patients’ tumour samples, indicating viral delivery can induce a successful immune response without causing any harm to the patient [49]. While the use of OVT remains very much at its infancy in the NSCLC setting, it will be interesting to see how this develops in the future. 

## 2. Immunotherapy for Advanced Stage NSCLC

In 2012, a phase I study was conducted to determine the safety and efficacy of nivolumab in patients with certain types of cancers. Surprisingly, this trial showed a response rate of 18% among patients with NSCLC and led to further clinical development and other anti-PD(L)1 agents in NSCLC [52]. Checkmate-017 was a landmark phase III trial that examined the safety and efficiency of nivolumab versus docetaxel. The trial showed significantly improved overall survival (OS), overall response rate (ORR), and progression-free survival (PFS) with nivolumab over docetaxel in patients who had received one prior line of therapy [21]. In March 2015, Checkmate-017 together with Checkmate-057, which had the same study design but was conducted in patients with non-squamous NSCLC [53], led to the approval by the FDA of nivolumab as the first ICI for the treatment of patients with advanced NSCLC after platinum-based chemotherapy. The five year outcomes of pooled data from both these trials revealed that at five years, nivolumab continued to demonstrate a survival benefit compared with docetaxel, with an OS of 13.4% versus 2.6%, and a PFS of 8.0% versus 0% [54]. This is a significant and life-changing advance for patients with NSCLC.

The approval of nivolumab was quickly followed by the approval of subsequent ICIs (Table 1). In October 2016, results from the KEYNOTE-010 [23] and KEYNOTE-024 [22] trials led to the approval of the PD-1 inhibitor, pembrolizumab, for the treatment of patients with metastatic NSCLC. In KEYNOTE-024, pembrolizumab was compared with cytotoxic chemotherapy as a first-line treatment for patients with advanced NSCLC and a PD-L1 tumour percentage score of 50% or greater. Pembrolizumab was shown to have significantly longer PS and OS and fewer adverse effects compared with platinum-based chemotherapy and so became a new standard of care for the first-line treatment of patients with ‘PD-L1 high’ NSCLC [22]. Following positive results from the POPLAR and OAK trials, another ICI inhibitor, atezolizumab, was approved for second-line treatment for patients with locally advanced or metastatic NSCLC [24,25]. While anti-PD-(L)1 monotherapy had demonstrated benefit in a subset of patients with pre-treated NSCLC, it was also postulated that combination immunotherapy may be a useful strategy in NSCLC.

Ipilimumab and tremelimumab are CTLA-4 inhibitors previously used in metastatic melanoma. In a phase II trial conducted more than 10 years ago, single agent ipilimumab combined with chemotherapy demonstrated modest benefit compared with ipilimumab monotherapy in NSCLC [55]. However, the combination of ipilimumab and nivolumab had demonstrated deep and durable responses in patients with melanoma [56], and it was hoped this could be recapitulated in NSCLC. In early phase studies of this combination, it was clear that the doses used for melanoma resulted in unacceptable toxicity in patients with NSCLC. After modification of this dose to ipilimumab every 6 weeks, the Checkmate-227 explored this combination in a complex eight-arm clinical trial. Broadly, this study demonstrated a significant benefit in PFS and OS as well as durable responses in patients with both PD-L1 > 1% and PD-L1 < 1% NSCLCs, and is approved as a ‘chemotherapy-free’ option in the first-line treatment for patients with advanced PD-L1 > 1% NSCLC [27]. In addition, a novel regimen from the Checkmate 9LA trial incorporating two doses of platinum-doublet chemotherapy in addition to ipilimumab and nivolumab also resulted in both PFS and OS benefits for this combination compared with chemotherapy alone, and is also an approved treatment option for first-line advanced NSCLC in all-comers for PD-L1 status [57]. This regimen is thought to potentially provide the ‘neoantigen release’ of cytotoxic chemotherapy alongside the long-term durable outcomes seen with ipilimumab and nivolumab.

While the use of ICIs has undoubtably changed the landscape of patient care for NSCLC, there remains an urgent need to transform more patients from immunotherapy non-responders to responders. Hence, current research strategies are focused on improving the response rate of ICIs, and these efforts are mainly centred around creating effective combination regimens with chemotherapy, radiotherapy, and other anti-cancer drugs (Table 4). In 2018, the FDA approved the use of pembrolizumab in combination with chemotherapy for first-line treatment of metastatic non-squamous NSCLC, independent of PD-L1 tumour expression status. This approval was based on the results of the KEYNOTE-189 trial which demonstrated a longer OS and PFS in patients receiving pembrolizumab in addition to standard chemotherapy of pemetrexed and a platinum-based drug compared with patients receiving chemotherapy alone [58]. This combination regimen was also approved for first-line treatment in metastatic squamous NSCLC following the results of the KEYNOTE-407 trial, again independent of PD-LI tumour expression status [59]. The positive results from the Impower150 trial led to the approval of atezolizumab with chemotherapy and bevacizumab for first-line treatment of metastatic non-squamous NSCLC [60]. Interestingly, it has been demonstrated that the combination of chemotherapy with bevacizumab induces proliferation of peripheral CD8 T cells, particularly memory and effector subsets [61], perhaps offering the rationale for combining chemotherapy and bevacizumab with ICIs. This is also now an approved option for first-line treatment of advanced NSCLC independent of PD-L1 status, based on the phase III IMPower150 trial [60]. 

Immunotherapy and immunotherapy combination strategies are now an accepted standard of care, with 5-year survival data supporting these approaches. The future of immunotherapy research in this disease setting will focus on improving treatment for subsets for patients with advanced disease, developing approaches for the PD-L1 pre-treated setting, and conducting biomarker discovery of the mechanisms of response and resistance.

## 3. Immunotherapy in the Treatment of Earlier Stage NSCLC 

While immunotherapy has changed the management of advanced NSCLC, its use in early-stage NSCLC has only begun to emerge. In February 2018, the FDA approved the use of durvalumab for patients with unresectable stage III NSCLC whose disease had not progressed following concurrent platinum-based chemotherapy and radiation therapy. This was the first approval of an immunotherapy agent for the treatment of earlier stage NSCLC and was based on the results of the phase III PACIFIC trial, which demonstrated prolonged PFS and OS in patients treated with 1 year of consolidation durvalumab versus a placebo [26]. In recent years, much effort has also centred around designing effective uses of immunotherapy for the treatment of resectable NSCLC (Table 5).

In October 2021, the FDA approved atezolizumab for adjuvant treatment following resection and platinum-based chemotherapy in patients with stage II and IIIA NSCLC whose tumours have a PD-L1 expression of greater than/equal 1% of tumour cells. This approval came about following the Impower010 trial which demonstrated a DFS benefit with atezolizumab versus best supportive care after adjuvant chemotherapy [102]. This was the first phase III trial to demonstrate a benefit from immunotherapy in patients with early-stage resectable NSCLC. In 2023, the KEYNOTE-091/PEARLS trial investigated pembrolizumab for adjuvant treatment of early-stage NSCLC following resection and optional platinum-based chemotherapy for patients with stage IB-IIIA NSCLC. This is approved for this indication, independent of tumoural PD-L1 expression [103].

**Table 5:** Clinical trials investigating ICIs in resectable NSCLC.

Drug Name	Additional Drug/Treatment	NCT/EU Identifier	Status	Trial Name	Phase	Reference
Nivolumab	Ipilimumab	NCT02259621	Recruiting	NA_00092076	Phase II (neoadjuvant)	[104]
	Ipilimumab	NCT03158129	Active, not recruiting	NEOSTAR	Phase II	[105]
	Platinum based-Chemotherapy	NCT02998528	Active, not recruiting	CheckMate 816	Phase III (neoadjuvant)	[106]
	Neoadjuvant Chemotherapy	NCT03081689	Active, not recruiting	NADIM phase II trial	Phase II (neoadjuvant)	[107]
	Neoadjuvant Chemotherapy	NCT04025879	Active, not recruiting	Checkmate 77T	Phase III (neoadjuvant)	[108]
Pembrolizumab		NCT03197467	Active, not recruiting	NEOMUN	Phase II (neoadjuvant)	[109]
		NCT02504372	Active, not recruiting	KEYNOTE-091/PEARLS	Phase II (neoadjuvant)	[109]
Ipilimumab	Chemotherapy	NCT01820754	Completed	TOP1201 IPI	Phase II (neoadjuvant)	[110]
Durvalumab	Neoadjuvant chemotherapy	NCT02572843	Active, not recruiting	SAKK 16/14	Phase II (adjuvant and neoadjuvant)	[111]
		NCT03030131	Terminated	IoNESCO trial	Phase II (neoadjuvant)	[112]
	Chemotherapy Oleculumab/ monailiziumab/ danvatirsen	NCT03800134 NCT03794544	Active, not recruiting Completed	AEGEAN Trial NEOCOAST	Phase III (adjuvant and neoadjuvant) Phase II (Neoadjuvant)	[113] [114]
Atezolizumab	Platinum-based chemotherapy	NCT02486718	Active, not recruiting	Impower010	Phase III (adjuvant)	[102]
		NCT02927301	Active, not recruiting		Phase II	[115]

We have also recently witnessed the use of immunotherapy in the neoadjuvant setting for NSCLC. The Checkmate-816 trial was a phase III trial examining the use of nivolumab plus platinum-based chemotherapy versus chemotherapy alone, followed by resection in patients with stage IB to IIIA resectable NSCLC; patients were enrolled regardless of PD-L1 status [112]. The results demonstrated that nivolumab plus chemotherapy resulted in significantly longer event-free survival and a higher percentage of patients with a pathological complete response than chemotherapy alone. Similar results have also been observed in the NADIM trial, for patients with resectable stage IIIA NSCLC [113]. The trial compares the effect of nivolumab with chemotherapy against chemotherapy as a monotherapy. While the trial is still ongoing, current results show an improved overall survival in patients treated with chemo-immunotherapy compared with chemotherapy alone, with overall survival at 24 months showing 85.3% vs. 64.8%.

In March 2022, the Checkmate-816 data led to the FDA approval of neoadjuvant nivolumab and platinum-doublet chemotherapy for the treatment of early-stage resectable NSCLC. The NEOSTAR trial was also performed in the neoadjuvant setting, examining the effect of nivolumab or nivolumab and ipilimumab followed by surgery in patients with resectable NSCLC. The data from this trial indicate that neoadjuvant nivolumab and ipilimumab-based therapy enhances pathological response, tumour immune infiltrates and immunological memory [111].

For patients with resectable NSCLC, the goal of therapy is cure. In the context of a curable disease, ICIs are used to reduce the risk of relapse, but it is critical that they do not interfere with the curative portion of the treatment paradigm, that is, surgery. This is a particular challenge for patients being treated in the neoadjuvant setting (e.g., CHECKMATE-816) in which the use of neoadjuvant therapy could be associated with delays to surgery. Encouragingly, an increased risk of surgical complications has not been observed in most neoadjuvant studies involving ICIs and NSCLC. For example, in the CHECKMATE-816 study, the authors reported surgical complications of 41.6% in the nivolumab plus chemotherapy arm and 46.7% in the chemotherapy arm [116]. Encouragingly, only 3.4% of patients had delayed surgery in the nivolumab plus chemotherapy arm and 5.1% in the chemotherapy alone arm. 

For patients in the adjuvant setting (e.g., PEARLS/IMPower010), different challenges emerge compared with the neoadjuvant setting [102,117]. Given that the cancer is already surgically resected, irAEs will not interfere with patients’ curative procedure. However, unlike in the metastatic setting, it is our expectation that the majority of these patients will be cured of their disease. In this regard, the risk of inducing a chronic or multi-organ toxicity may be associated with significant and long-term impact on patients’ quality-of-life. The risks and benefits of such adjuvant therapy pose challenges in weighing the low risk of long-term toxicity versus the survival benefit of disease control. Decisions regarding the most appropriate treatment option need to be made in a collaborative manner between patients and the multidisciplinary team.

Adjuvant/neoadjuvant studies in resectable NSCLC have to date demonstrated impressive event-free/disease-free survival for patients, which we hope will translate to an overall survival benefit. Long-term follow-up and further studies will provide more data on the efficacy of adjuvant/neoadjuvant ICI and the risks of delays to surgical resection/long-term toxicity. We eagerly await the publication of further studies in this field and long-term follow-up.

## 4. Challenges Associated with the Use of Immunotherapy 

### 4.1. Immune-Related Adverse Events (irAEs)

Despite the clinical benefit that arises from immunotherapy, more than 20% of patients experience immune-related adverse events (irAEs) from therapy, and the incidence may be >50% with combination approaches [118]. Immune-related adverse events can be described as autoimmune conditions that can affect any organ system in the body after ICI administration [119]. These toxicities have presented as a challenge for clinical practitioners and patients because rather than managing familiar side effects such as nausea, anaemia, and immunosuppression, they are now confronted with unfamiliar side effects, such as underactive pituitary glands and hepatitis [119]. Immune-related adverse events vary in terms of their onset time, severity, and underlying biology [120]. They can affect a broad range of organs and occur at any time during the patient’s treatment course. They most commonly occur in the first three months of treatment but have also been observed to occur long after ICI has been stopped [121]. The time of irAEs occurrence gives an indication of the severity of the effects on the patient. For example, toxicity in the first year of therapy strongly correlated with long term toxicity beyond 1 year [120]. Many ICI clinical trials have reported longer-term safety data, but information is still limited about the ongoing impact of the toxicities [121]. Treatments for irAEs centre around glucocorticoids for acute irAEs (developed during ICI treatment) specifically, with good effects observed after several weeks. Although most irAEs resolve, some develop into a chronic state (develop after ICI treatment has terminated) and lifelong therapy such as hormonal supplementation or immunosuppression may be required [119].

IrAEs are distinct from those that occur with traditional chemotherapy or other forms of anti-cancer therapy as they occur as a result of the immunologic mode of action of ICIs. There are limited data exploring the mechanisms that underpin the development of irAEs. However, T-cell [122,123,124], B-cell [125] and macrophage-related mechanisms [123] have been identified. In a comprehensive clinical and translational study, the cytokine interleukins-6 (IL-6) was shown to be highly upregulated in the patient cohort after nivolumab treatment [124]. Moreover, blocking IL-6 in mouse models could potentially mitigate autoimmunity and maintain, or even possibly boost, the tumour immunity [124]. Recently, it was observed that the use of IL-6 blockade in a patient with ICI-induced irAEs led to successful mitigation of irAEs symptoms without compromising the ICI treatment [124]. It has been demonstrated that changes in T cell populations occur early after ICI treatment [122], and these changes can also affect B cells and macrophages directly or indirectly; however, the mechanism for this remains largely unknown [125]. Early changes in B cells have also correlated with high rates of irAEs, indicating B cells may play an important role in driving irAEs [125]. The link between T cells, B cells, and macrophages in relation to irAEs and the modes of actions they employ, is of major interest of the immunotherapy field at present. 

It is critical that effective strategies are developed in the clinic to address the issue of irAEs associated with ICIs. Clinical experience of ICI toxicity develops local expertise in managing the diverse range of potential irAEs associated with ICIs. In recent years, there has been the publication of international guidelines for the management of irAEs to guide clinicians in the clinical management of these complex cases. These include the National Comprehensive Cancer Network (NCCN) and the European Society of Medical Oncology (ESMO) guidelines [126,127]. These provide detailed decision assistance tools regarding risk stratification, early diagnosis, steroid administration and steroid sparing strategies for patients experiencing irAEs.

The management of irAEs ideally involves specialist teams involving a medical oncologist and an organ specialist (e.g., a respiratory physician in case of pneumonitis). In some institutions, this has been formalised into a dedicated irAE toxicity team. This form of multidisciplinary team has demonstrated feasibility and been shown to change patient management [128]. 

Critical areas of research include the appropriate risk stratification of patients and the development of biomarkers for early identification of irAEs. Prior to treatment initiation, risk stratification of those at high risk of irAEs is a critical step in ensuring patients are not exposed to an unacceptable level of risk. For example, it has been demonstrated that patients with a history of interstitial lung disease are at high-risk of developing ICI associated pneumonitis [129]. In the case of cardiotoxicity, prospective data would suggest that baseline ECG/troponin can be helpful in identifying those patients most at risk of toxicity, and these patients may warrant close surveillance. It is likely that the future of ICI toxicity management will involve a refinement of our strategies to identify those patients most at risk through clinical studies and biomarker identification in tandem with improvements in immunosuppressive strategies.

### 4.2. Biomarkers 

The reasons underlying why some patients with NSCLC achieve disease control from therapy, or develop toxicity, are incompletely understood. Hence, another major challenge facing the modern era of immunotherapy treatment lies in the development of efficient biomarkers to optimize patient selection. The expression of PD-L1 on tumour cells, quantified using IHC, is currently the most widely used and validated biomarker to guide the selection of patients to receive ICIs. PD-L1 expression has shown predictive value in many clinical trials in NSCLC, with correlations observed between clinical response and increased expression of PD-L1 on tumour cells [23,24,130,131]. However, positive correlation of PD-L1 expression can only partially predict which patients will benefit from ICIs, and many trials have demonstrated responses irrespective of PD-L1 expression status [5,21]. This imperfection in the use PD-L1 as a biomarker of response could be attributed to many factors. For instance, differences exist in the specific types of assays that are utilized to assess PD-L1 expression in tumour tissues, and even within these assays it can prove difficult to score the PD-L1 expression consistently and accurately on tumour cells and immune cells [132]. Moreover, intra-tumour heterogeneity (ITH) exists within the tumour of patients with NSCLC, and hence, the biopsy samples may not accurately reflect the expression of PD-L1 throughout the tumour [133,134]. The emerging use of liquid biopsies to assess the expression of PD-L1 using cytology samples has shown great promise, and so may help overcome some of these challenges in the future [39]. 

Tumour mutational burden (TMB) refers to the absolute number of non-synonymous mutations within a tumour, which leads to the generation of immunogenic neo-peptides displayed on the surface of tumour cells, and hence, is associated with a greater CD8^+^ T cell response following ICI treatment [135]. In June 2020, the FDA approved the use of pembrolizumab for the treatment of unresectable and metastatic solid tumours with a high TMB. This approval was based on the results of the KEYNOTE-158 trial which demonstrated that a high TMB was associated with an increased objective response rate [136]. However, similar to the use of PD-L1 expression, the predictive value of TMB is limited by the presence of ITH. A high ITH may result in the neoantigens only being present on a subset of cells and hence, the immune response may not be effective against the entire tumour [137]. Moreover, sub-clonal neoantigens, which occur as a result of cytotoxic-chemotherapy, give rise to high TMB and these sub-clonal neoantigens are associated with poor responders. This is in comparison to an enhanced response to ICI in patients with tumours enriched for clonal neoantigens [138]. Hence, neoantigen ITH can also contribute to the limited predictive value of TMB, emphasizing the need for effective diagnostic techniques that examine the entire tumour.

Another biomarker of response which is currently gaining much attention in the field of NSCLC is the use of circulating tumour DNA (ctDNA). Cells release small double-stranded DNA fragments into the bloodstream during apoptosis and necrosis, termed circulating free DNA (cfDNA), and in cancer patients, small fractions of cfDNA can be shed from the tumour in the form of ctDNA [139]. Several studies have demonstrated that the levels of ctDNA, as detected by a liquid biopsy, can predict response to ICI in patients with NSCLC [140,141,142]. Moreover, ctDNA can be used for the detection of point mutations associated with sensitivity to ICI. For example, several studies have demonstrated that mutations in STK11 can predict the response to treatment [143], including ICIs [144] in NSCLC. Other biomarkers assessed by liquid biopsy which may offer potential in predicting the response to ICI in the future include peripheral blood cytokines [145], circulating non-coding RNA [146] and the levels of various immune cell populations [147,148,149]. 

Finally, biomarkers to predict irAEs are even less characterized. Some research suggests the composition of the gut microbiome in response to ICI can influence the development of irAEs [150,151]; however, the mechanism of action remains largely unclear. Other potential irAEs biomarkers include baseline auto-antibodies [152], germline genetics [153], T cell and B cell populations [133], and shared T cell antigens [132]. Given the adverse effects that ICI can have on patients, it is imperative that the field invests research in developing predictive biomarkers to optimise patient selection and deliver this therapy to patients most likely to benefit.

## 5. Future Outlook for the Use of Immunotherapy in NSCLC

ICIs have established efficacy in advanced and early-stage NSCLC, and it is unlikely that PD-1/PD-L1 monotherapy will improve on the already established benefit for patients. As mentioned previously in this review, recent interest lies in combining ICIs with novel combination strategies which could include radiation, novel ICIs or other novel systemic therapies. For example, neoadjuvant durvalumab was investigated with or without sub-ablative stereotactic radiotherapy (SBRT) in patients with resectable NSCLC [154]. In this study patients received two cycles of neoadjuvant durvalumab +/− 3 fractions of SBRT (8 Gy * 3), followed by surgery. In the NEOCOAST study, the authors investigated neoadjuvant durvalumab +/− novel agents for resectable stage I–IIIA NSCLC [155]. These novel agents included the anti-CD73 agent oleclumab, the anti-NKG2A monalizumab or the anti-STAT3 antisense oligonucleotide danavatirsen. Finally, the inhibition of novel checkpoints as a monotherapy or in combination with pre-existing ICIs is an emerging strategy in this field. LAG-3 suppresses T cell activation and cytokine secretion [156]. Inhibitors of LAG-3 have already reached phase II and phase III clinical trials and relatlimab has been approved in combination with nivolumab for advanced melanoma [157]. In NSCLC, the RELATIVITY study has commenced accrual and is exploring the combination of relatlimab and nivolumab and chemotherapy in the advanced setting [158].

Another critical area of research in this field is utilising ‘liquid biopsy’ assays to identify patients not responding to therapy at an early stage in their treatment paradigm so that therapy plans can be adapted thereafter. ‘Liquid Biopsy’ generally refers to the use of blood based (but can use other body fluids, e.g., breath) biomarkers to identify tumour-based signatures which could include cfDNA, ctDNA, circulating tumour cells (CTCs) and others [159]. In an investigation of 67 patients with stage IV NSCLC, a ctDNA ‘molecular response’ in plasma 9 weeks post starting ICI was associated with a durable clinical benefit (defined as an ongoing response at 6 months post IO, 3.5% vs. 49.4%, *p* < 0.001). This study demonstrates the potential for ctDNA dynamics to identify responders to therapy.

In summary, it is likely that the future of ICIs in NSCLC will involve their combination with other systemic/local therapy but advances in therapeutics will be combined with novel diagnostics/biomarkers (e.g., liquid biopsy) to improve patient selection for therapy.

## 6. Conclusions

The introduction of immunotherapy as a treatment option for patients with NSCLC has offered benefit and hope to selected patients. This is reflected in the survival benefit and improvements in the quality of life for these patients. However, the subset of patients who sustain a prolonged anti-tumour response remains relatively low. Continued development of effective immunotherapy-based combination regimens and expansion into earlier stage NSCLC will hopefully increase the proportion of patients who respond to ICIs in the future. Moreover, a critical gap in the field is to develop predictive biomarkers to identify patients who will benefit most from ICI or develop toxicity. The identification of such biomarkers and their integration with clinical care and therapeutic decision-making would continue to ensure the impact of immunotherapy for NSCLC in the future.

## Figures and Tables

**Table 1:** Immune checkpoint inhibitors approved for the treatment of NSCLC.

Name	Approval	Type of MA	Action	Usage	Reference
Nivolumab	March 2015	IgG4	PD-1	Stage III OR IV metastatic NSCLC.	[21]
Pembrolizumab	October 2016	Humanized IgG4-K isotope antibody.	PD-1	Stage IV metastatic NSCLC.	[22,23]
Atezolizumab	October 2016	IgG1	PD-L1	Stage III or IV metastatic NSCLC.	[24,25]
Durvalumab	February 2018	IgG1 k	PD-L1	Stage III NSCLC	[26]
Ipilimumab	May 2020 (in combination with nivolumab)	IgG1	CTLA-4	NSCLC	[27]
Cemiplimab	November 2022 (in combination with platinum-based chemotherapy)	IgG4	PD-L1	Stage III OR IV metastatic NSCLC	[28]
Tremelimumab	November 2022 (in combination with durvalumab and platinum-based chemotherapy)	IgG2	PD-L1	Stage III OR IV metastatic NSCLC	[29]

**Table 2:** Clinical trials investigating CAR-T cell therapy in NSCLC.

ClinicalTrials.gov Identifier	Status	Phase	Target	Cancer Type
NCT04153799	Unknown	Phase I	EGFR	NSCLC
NCT05060796	Recruiting	Early Phase I	EGFR	NSCLC
NCT03525782	Unknown	Phase I/II	MUC1	NSCLC
NCT04489862	Unknown	Early Phase I	MSLN	NSCLC
NCT03060343	Unknown	Early Phase I	PD-L1 CD80/CD86	NSCLC
NCT04556669	Recruiting	Phase I	CD22	NSCLC
NCT05620342	Not yet recruiting	Early Phase I	GD2	NSCLC
NCT05117138	Not yet recruiting	Phase I/II	AMT-116	NSCLC
NCT02587689	Unknown	Phase I/II	MUC1	NSCLC
NCT05274451	Recruiting	Phase I	ROR1	NSCLC
NCT04981119	Recruiting	-	HLA negative	NSCLC
NCT02706392	Terminated	Phase I	ROR1	NSCLC
NCT04025216	Active, not recruiting	Phase I	MUC1	NSCLC
NCT05239143	Recruiting	Phase I	MUC1	NSCLC
NCT03182816	Unknown	Phase I/II	CTLA-4/PD-1	Malignant solid tumours
NCT03932565	Unknown	Phase I	Nectin4/FAP	NSCLC
NCT03638206	Recruiting	Phase I/II	Multi-target	Lung cancer
NCT03740256	Recruiting	Phase I	HER-2	Lung cancer
NCT03198052	Recruiting	Phase I	Multi-target (PSCA, MUC1, TGFβ, HER2, Mesothelin, Lewis-Y, GPC3, AXL, EGFR, Claudin18.2, or B7-H3)	Lung cancer
NCT03356808	Unknown	Phase I/II	Multi-target (MAGE-A1, MAGE-A4, MucI, GD2, and mesothelin, as well as novel cancer antigens)	Lung cancer
NCT03054298	Recruiting	Phase I	Mesothelin	Lung Adenocarcinoma
NCT03198546	Recruiting	Phase I	GPC3 and/or TGFβ	Squamous Cell Lung Cancer
NCT02992210	Unknown	Phase I/II		Solid tumour
NCT02349724	Unknown	Phase I	CEA	Lung cancer
NCT02414269	Active, not recruiting	Phase I/II	Mesothelin	Lung cancer
NCT01869166	Unknown	Phase I/II	EGFR	NSCLC
NCT01583686	Terminated	Phase I/II	Mesothelin	Lung cancer

**Table 3:** Clinical trials investigating OVT in NSCLC.

ClinicalTrials.gov Identifier	Status	Phase	Virus	Cancer Type
NCT03004183	Active, not recruiting	Phase II	ADV/HSV-tk	NSCLC
NCT05076760	Recruiting	Phase I	MEM-288	NSCLC
NCT02879760	Completed	Phase I/II	Ad-MAGEA3 and MG1-MAGEA3	NSCLC
NCT02053220	Completed	Phase I	Colo-Ad1	NSCLC
NCT05602792	Recruiting	Phase I/II	T3011	NSCLC
NCT00861627	Completed	Phase II	REOLYSIN^®^	NSCLC
NCT03767348	Recruiting	Phase II	RP1	NSCLC
NCT04725331	Recruiting	Phase I/II	BT-001	NSCLC

**Table 4:** Selected clinical trials investigating combination strategies with approved ICIs in advanced NSCLC.

**Ipilimumab**						
**Additional** **Treatment**	**Drug Type**	**NCT/EU Identifier**	**Trial Name**	**Phase**	**Status**	**Reference**
Chemotherapy	Paclitaxel, Carboplatin	NCT01285609		Phase III	Completed	[62]
Other	Radiotherapy	NCT02221739		Phase I/II	Completed	[63]
	Erlotinib or Crizotinib	NCT01998126		Phase I	Completed	[64]
**Cemiplimab**						
**Additional** **Treatment**	**Drug Type**	**NCT/EU Identifier**	**Trial Name**	**Phase**	**Status**	**Reference**
Chemotherapy	Platinum-doublet chemotherapy	NCT03409614	EMPOWER-Lung 3	Phase III	Active, not recruiting	[28]
Other	Isatuximab	NCT03367819		Phase I/II	Terminated	[65]
**Nivolumab**						
**Additional Treatment**	**Drug Type**	**NCT/EU Identifier**	**Trial Name**	**Phase**	**Status**	**Reference**
Chemotherapy	Platinum-doublet chemotherapy	NCT01454102	CheckMate 012	Phase I	Completed	[66]
	Ipilimumab plus chemotherapy	NCT03215706	CheckMate 9LA	Phase III	Active, not recruiting	[67]
	Platinum-doublet chemotherapy	NCT02477826	CheckMate 227	Phase II	Active, not recruiting	[27]
	Veliparib, and platinum-doublet chemotherapy	NCT02944396		Phase I	Completed	[68]
Immune Checkpoint Inhibitors	Ipilimumab	NCT01454102	CheckMate 012	Phase I	Completed	[69]
		NCT02659059	CheckMate 568	Phase II	Completed	[70]
		NCT02785952	Lung-MAP S1400I	Phase III	Active, not recruiting	[71]
		NCT02477826	Checkmate 227	Phase III	Active, not recruiting	[27]
Vaccines	CV301	NCT02840994		Phase I	Completed	[72]
	NEO-PV-01	NCT02897765		Phase I	Completed	[73]
**Durvalumab**						
**Additional Treatment**	**Drug Type**	**NCT/EU Identifier**	**Trial Name**	**Phase**	**Status**	**Reference**
Immune Checkpoint Inhibitors	Tremelimumab	NCT02000947		Phase I	Completed	[5]
		NCT03373760		Phase II	Completed	[74]
	Tremelimumab +/− chemotherapy	NCT03057106		Phase II	Active, not recruiting	[75]
Anti-Angiogenic	Ramucirumab	NCT02572687		Phase Ia/b	Completed	[76]
Other	Gefitinib	NCT02088112		Phase I	Completed	[77]
	LY3022855	NCT02718911		Phase Ia/b	Completed	[78]
	AZD4635	NCT02740985		Phase I	Active, not recruiting	[79]
	Osimertinib	NCT02454933	CAURAL	Phase III	Active, not recruiting	[80]
**Atezolizumab**						
**Additional** **Treatment**	**Drug Type**	**NCT/EU Identifier**	**Trial Name**	**Phase**	**Status**	**Reference**
Chemotherapy	Carboplatin and paclitaxel or carboplatin and nab-paclitaxel	NCT02367794	iMpower131	Phase III	Completed	[81]
	Vinorelbine	NCT03801304	VinMetAtezo	Phase II	Completed	[82]
	Carboplatin and paclitaxel with bevacizumab	NCT02366143	iMpower150	Phase III	Completed	[60]
	Carboplatin and nab-paclitaxel	NCT02367781	iMpower130	Phase III	Completed	[83]
	Pemetrexed and either cisplatin or carboplatin	NCT02657434	iMpower132	Phase III	Completed	[84]
Immune Checkpoint Inhibitors	Ipilimumab	NCT02174172		Phase Ib	Completed	[85]
Other	Navoximod	NCT02471846		Phase I	Completed	[86]
**Pembrolizumab**						
**Additional Treatment**	**Drug Type**	**NCT/EU Identifier**	**Trial Name**	**Phase**	**Status**	**Reference**
Chemotherapy	Chemotherapy agents	NCT02039674	KEYNOTE-021	Phase I/II	Completed	[87]
		NCT01840579	KEYNOTE-011	Phase I	Completed	[88]
	Pemetrexed/platinum chemotherapy	NCT02578680	KEYNOTE-189	Phase III	Active, not recruiting	[89]
	Docetaxel	NCT02574598		Phase II	Completed	[90]
Immune Checkpoint Inhibitors	Ipilimumab	NCT02039674	KEYNOTE-021	Phase I/II	Completed	[87]
Anti-Angiogenic	Ramucirumab	NCT02443324		Phase I	Completed	[91]
		NCT03971474		Phase II	Active, not recruiting	[92]
	Lenvatinib	NCT02501096		Phase I/II	Completed	[93]
		NCT03006887		Phase I	Completed	[94]
Other	Pegilodecakin	NCT02009449		Phase I	Active, not recruiting	[95]
	Necitimumab	NCT02451930		Phase I	Completed	[96]
	Oral Azacitidine	NCT02546986		Phase II	Active, not recruiting	[97]
	Afatinib	NCT03157089	LUX-Lung-IO	Phase II	Completed	[98]
	Eprenetapopt	NCT04383938		Phase I/II	Completed	[99]
	Niraparib	NCT04475939	JASPER	Phase II	Active, not recruiting	[100]
	Stereotactic body radiotherapy (SBRT)	NCT02608385		Phase I	Active, not recruiting	[101]

## References

[1] Cascone T., Fradette J., Pradhan M., Gibbons D.L. (2022). Tumor Immunology and Immunotherapy of Non-Small-Cell Lung Cancer. Cold Spring Harb. Perspect. Med..

[2] Buchbinder E.I., Desai A. (2016). CTLA-4 and PD-1 pathways: Similarities, differences, and implications of their inhibition. Am. J. Clin. Oncol..

[3] Berghmans T., Durieux V., Hendriks L.E., Dingemans A.-M. (2020). Immunotherapy: From advanced NSCLC to early stages, an evolving concept. Front. Med..

[4] Tang S., Qin C., Hu H., Liu T., He Y., Guo H., Yan H., Zhang J., Tang S., Zhou H. (2022). Immune Checkpoint Inhibitors in Non-Small Cell Lung Cancer: Progress, Challenges, and Prospects. Cells.

[5] Antonia S., Goldberg S.B., Balmanoukian A., Chaft J.E., Sanborn R.E., Gupta A., Narwal R., Steele K., Gu Y., Karakunnel J.J. (2016). Safety and antitumour activity of durvalumab plus tremelimumab in non-small cell lung cancer: A multicentre, phase 1b study. Lancet Oncol..

[6] Casey S.C., Tong L., Li Y., Do R., Walz S., Fitzgerald K.N., Gouw A.M., Baylot V., Gütgemann I., Eilers M. (2016). MYC regulates the antitumor immune response through CD47 and PD-L1. Science.

[7] Ding X.-C., Wang L.-L., Zhang X.-D., Xu J.-L., Li P.-F., Liang H., Zhang X.-B., Xie L., Zhou Z.-H., Yang J. (2021). The relationship between expression of PD-L1 and HIF-1α in glioma cells under hypoxia. J. Hematol. Oncol..

[8] Green M.R., Monti S., Rodig S.J., Juszczynski P., Currie T., O’Donnell E., Chapuy B., Takeyama K., Neuberg D., Golub T.R. (2010). Integrative analysis reveals selective 9p24. 1 amplification, increased PD-1 ligand expression, and further induction via JAK2 in nodular sclerosing Hodgkin lymphoma and primary mediastinal large B-cell lymphoma. Blood J. Am. Soc. Hematol..

[9] Micevic G., Thakral D., McGeary M., Bosenberg M.W. (2019). PD-L1 methylation regulates PD-L1 expression and is associated with melanoma survival. Pigment. Cell Melanoma Res..

[10] Amini M., Hejazi M., Ghorban K., Mokhtarzadeh A., Baradaran B. (2021). Identification of functional methylated CpG loci in PD-L1 promoter as the novel epigenetic biomarkers for primary gastric cancer. Gene.

[11] Lim S.-O., Li C.-W., Xia W., Cha J.-H., Chan L.-C., Wu Y., Chang S.-S., Lin W.-C., Hsu J.-M., Hsu Y.-H. (2016). Deubiquitination and stabilization of PD-L1 by CSN5. Cancer Cell.

[12] Hsu J.-M., Xia W., Hsu Y.-H., Chan L.-C., Yu W.-H., Cha J.-H., Chen C.-T., Liao H.-W., Kuo C.-W., Khoo K.-H. (2018). STT3-dependent PD-L1 accumulation on cancer stem cells promotes immune evasion. Nat. Commun..

[13] Cha J.-H., Yang W.-H., Xia W., Wei Y., Chan L.-C., Lim S.-O., Li C.-W., Kim T., Chang S.-S., Lee H.-H. (2018). Metformin promotes antitumor immunity via endoplasmic-reticulum-associated degradation of PD-L1. Mol. Cell.

[14] Zhou Z., Liu Y., Song W., Jiang X., Deng Z., Xiong W., Shen J. (2022). Metabolic reprogramming mediated PD-L1 depression and hypoxia reversion to reactivate tumor therapy. J. Control. Release.

[15] Liu Y., Zhou Z., Hou J., Xiong W., Kim H., Chen J., Zheng C., Jiang X., Yoon J., Shen J. (2022). Tumor Selective Metabolic Reprogramming as a Prospective PD-L1 Depression Strategy to Reactivate Immunotherapy. Adv. Mater..

[16] Morrissey S.M., Zhang F., Ding C., Montoya-Durango D.E., Hu X., Yang C., Wang Z., Yuan F., Fox M., Zhang H.-G. (2021). Tumor-derived exosomes drive immunosuppressive macrophages in a pre-metastatic niche through glycolytic dominant metabolic reprogramming. Cell Metab..

[17] Franzin R., Netti G.S., Spadaccino F., Porta C., Gesualdo L., Stallone G., Castellano G., Ranieri E. (2020). The use of immune checkpoint inhibitors in oncology and the occurrence of AKI: Where do we stand?. Front. Immunol..

[18] De Giglio A., Di Federico A., Nuvola G., Deiana C., Gelsomino F. (2021). The landscape of immunotherapy in advanced NSCLC: Driving beyond PD-1/PD-L1 inhibitors (CTLA-4, LAG3, IDO, OX40, TIGIT, vaccines). Curr. Oncol. Rep..

[19] Reck M., Borghaei H., O’Byrne K.J. (2019). Nivolumab plus ipilimumab in non-small-cell lung cancer. Future Oncol..

[20] Menon T., Gopal S., Rastogi Verma S. (2022). Targeted therapies in non-small cell lung cancer and the potential role of AI interventions in cancer treatment. Biotechnol. Appl. Biochem..

[21] Brahmer J., Reckamp K.L., Baas P., Crinò L., Eberhardt W.E.E., Poddubskaya E., Antonia S., Pluzanski A., Vokes E.E., Holgado E. (2015). Nivolumab versus Docetaxel in Advanced Squamous-Cell Non–Small-Cell Lung Cancer. N. Engl. J. Med..

[22] Reck M., Rodríguez-Abreu D., Robinson A.G., Hui R., Csőszi T., Fülöp A., Gottfried M., Peled N., Tafreshi A., Cuffe S. (2016). Pembrolizumab versus chemotherapy for PD-L1–positive non–small-cell lung cancer. N. Engl. J. Med..

[23] Herbst R.S., Baas P., Kim D.-W., Felip E., Pérez-Gracia J.L., Han J.-Y., Molina J., Kim J.-H., Arvis C.D., Ahn M.-J. (2016). Pembrolizumab versus docetaxel for previously treated, PD-L1-positive, advanced non-small-cell lung cancer (KEYNOTE-010): A randomised controlled trial. Lancet.

[24] Fehrenbacher L., Spira A., Ballinger M., Kowanetz M., Vansteenkiste J., Mazieres J., Park K., Smith D., Artal-Cortes A., Lewanski C. (2016). Atezolizumab versus docetaxel for patients with previously treated non-small-cell lung cancer (POPLAR): A multicentre, open-label, phase 2 randomised controlled trial. Lancet.

[25] Rittmeyer A., Barlesi F., Waterkamp D., Park K., Ciardiello F., Von Pawel J., Gadgeel S.M., Hida T., Kowalski D.M., Dols M.C. (2017). Atezolizumab versus docetaxel in patients with previously treated non-small-cell lung cancer (OAK): A phase 3, open-label, multicentre randomised controlled trial. Lancet.

[26] Antonia S.J., Villegas A., Daniel D., Vicente D., Murakami S., Hui R., Yokoi T., Chiappori A., Lee K.H., de Wit M. (2017). Durvalumab after chemoradiotherapy in stage III non–small-cell lung cancer. N. Engl. J. Med..

[27] Hellmann M.D., Paz-Ares L., Bernabe Caro R., Zurawski B., Kim S.-W., Carcereny Costa E., Park K., Alexandru A., Lupinacci L., de la Mora Jimenez E. (2019). Nivolumab plus Ipilimumab in Advanced Non–Small-Cell Lung Cancer. N. Engl. J. Med..

[28] Gogishvili M., Melkadze T., Makharadze T., Giorgadze D., Dvorkin M., Penkov K., Laktionov K., Nemsadze G., Nechaeva M., Rozhkova I. (2022). Cemiplimab plus chemotherapy versus chemotherapy alone in non-small cell lung cancer: A randomized, controlled, double-blind phase 3 trial. Nat. Med..

[29] Johnson M.L., Cho B.C., Luft A., Alatorre-Alexander J., Geater S.L., Laktionov K., Kim S.W., Ursol G., Hussein M., Lim F.L. (2023). Durvalumab with or without Tremelimumab in Combination with Chemotherapy as First-Line Therapy for Metastatic Non-Small-Cell Lung Cancer: The Phase III POSEIDON Study. J. Clin. Oncol..

[30] Alemohammad H., Najafzadeh B., Asadzadeh Z., Baghbanzadeh A., Ghorbaninezhad F., Najafzadeh A., Safarpour H., Bernardini R., Brunetti O., Sonnessa M. (2022). The importance of immune checkpoints in immune monitoring: A future paradigm shift in the treatment of cancer. Biomed. Pharmacother..

[31] Zhu C., Anderson A.C., Kuchroo V.K. (2011). TIM-3 and its regulatory role in immune responses. Negative Co-Receptors and Ligands.

[32] Kouo T., Huang L., Pucsek A.B., Cao M., Solt S., Armstrong T., Jaffee E. (2015). Galectin-3 Shapes Antitumor Immune Responses by Suppressing CD8+ T Cells via LAG-3 and Inhibiting Expansion of Plasmacytoid Dendritic CellsGalectin-3 Regulates CD8+ T Cells via LAG-3 and pDCs. Cancer Immunol. Res..

[33] Yu X., Harden K., C Gonzalez L., Francesco M., Chiang E., Irving B., Tom I., Ivelja S., Refino C.J., Clark H. (2009). The surface protein TIGIT suppresses T cell activation by promoting the generation of mature immunoregulatory dendritic cells. Nat. Immunol..

[34] Huang X., Zhang X., Li E., Zhang G., Wang X., Tang T., Bai X., Liang T. (2020). VISTA: An immune regulatory protein checking tumor and immune cells in cancer immunotherapy. J. Hematol. Oncol..

[35] Desai A., Peters S. (2023). Immunotherapy-based combinations in metastatic NSCLC. Cancer Treat. Rev..

[36] Cho B.C., Abreu D.R., Hussein M., Cobo M., Patel A.J., Secen N., Lee K.H., Massuti B., Hiret S., Yang J.C.H. (2022). Tiragolumab plus atezolizumab versus placebo plus atezolizumab as a first-line treatment for PD-L1-selected non-small-cell lung cancer (CITYSCAPE): Primary and follow-up analyses of a randomised, double-blind, phase 2 study. Lancet Oncol..

[37] Creelan B.C., Wang C., Teer J.K., Toloza E.M., Yao J., Kim S., Landin A.M., Mullinax J.E., Saller J.J., Saltos A.N. (2021). Tumor-infiltrating lymphocyte treatment for anti-PD-1-resistant metastatic lung cancer: A phase 1 trial. Nat. Med..

[38] Cao W., Xing H., Li Y., Tian W., Song Y., Jiang Z., Yu J. (2022). Claudin18.2 is a novel molecular biomarker for tumor-targeted immunotherapy. Biomark. Res..

[39] Brozos-Vázquez E.M., Díaz-Peña R., García-González J., León-Mateos L., Mondelo-Macía P., Peña-Chilet M., López-López R. (2021). Immunotherapy in nonsmall-cell lung cancer: Current status and future prospects for liquid biopsy. Cancer Immunol. Immunother..

[40] Liu Y., Chen X., Han W., Zhang Y. (2017). Tisagenlecleucel, an approved anti-CD19 chimeric antigen receptor T-cell therapy for the treatment of leukemia. Drugs Today (Barc.).

[41] Li H., Harrison E.B., Li H., Hirabayashi K., Chen J., Li Q.X., Gunn J., Weiss J., Savoldo B., Parker J.S. (2022). Targeting brain lesions of non-small cell lung cancer by enhancing CCL2-mediated CAR-T cell migration. Nat. Commun..

[42] Reppel L., Tsahouridis O., Akulian J., Davis I.J., Lee H., Fucà G., Weiss J., Dotti G., Pecot C.V., Savoldo B. (2022). Targeting disialoganglioside GD2 with chimeric antigen receptor-redirected T cells in lung cancer. J. Immunother. Cancer.

[43] Jie Y., Liu G., Feng L., Li Y., E M., Wu L., Li Y., Rong G., Li Y., Wei H. (2021). PTK7-Targeting CAR T-Cells for the Treatment of Lung Cancer and Other Malignancies. Front. Immunol..

[44] Fan C., Qu H., Wang X., Sobhani N., Wang L., Liu S., Xiong W., Zeng Z., Li Y. (2021). Cancer/testis antigens: From serology to mRNA cancer vaccine. Semin. Cancer Biol..

[45] Leighl N.B., Hellmann M.D., Hui R., Carcereny E., Felip E., Ahn M.J., Eder J.P., Balmanoukian A.S., Aggarwal C., Horn L. (2019). Pembrolizumab in patients with advanced non-small-cell lung cancer (KEYNOTE-001): 3-year results from an open-label, phase 1 study. Lancet Respir. Med..

[46] Larson C., Oronsky B., Reid T. (2022). AdAPT-001, an oncolytic adenovirus armed with a TGF-β trap, overcomes in vivo resistance to PD-L1-immunotherapy. Am. J. Cancer Res..

[47] Lei W., Liu H.B., Wang S.B., Zhou X.M., Zheng S.D., Guo K.N., Ma B.Y., Xia Y.L., Tan W.S., Liu X.Y. (2013). Tumor suppressor in lung cancer-1 (TSLC1) mediated by dual-regulated oncolytic adenovirus exerts specific antitumor actions in a mouse model. Acta Pharmacol. Sin..

[48] Rudin C.M., Pandha H.S., Zibelman M., Akerley W.L., Harrington K.J., Day D., Hill A.G., O’Day S.J., Clay T.D., Wright G.M. (2023). Phase 1, open-label, dose-escalation study on the safety, pharmacokinetics, and preliminary efficacy of intravenous Coxsackievirus A21 (V937), with or without pembrolizumab, in patients with advanced solid tumors. J. Immunother. Cancer.

[49] Garcia-Carbonero R., Salazar R., Duran I., Osman-Garcia I., Paz-Ares L., Bozada J.M., Boni V., Blanc C., Seymour L., Beadle J. (2017). Phase 1 study of intravenous administration of the chimeric adenovirus enadenotucirev in patients undergoing primary tumor resection. J. Immunother. Cancer.

[50] Ripp J., Hentzen S., Saeed A. (2022). Oncolytic Viruses as an Adjunct to Immune Checkpoint Inhibition. Front. Biosci. (Landmark Ed.).

[51] Andtbacka R.H., Kaufman H.L., Collichio F., Amatruda T., Senzer N., Chesney J., Delman K.A., Spitler L.E., Puzanov I., Agarwala S.S. (2015). Talimogene Laherparepvec Improves Durable Response Rate in Patients with Advanced Melanoma. J. Clin. Oncol..

[52] Topalian S.L., Hodi F.S., Brahmer J.R., Gettinger S.N., Smith D.C., McDermott D.F., Powderly J.D., Carvajal R.D., Sosman J.A., Atkins M.B. (2012). Safety, activity, and immune correlates of anti-PD-1 antibody in cancer. N. Engl. J. Med..

[53] Borghaei H., Paz-Ares L., Horn L., Spigel D.R., Steins M., Ready N.E., Chow L.Q., Vokes E.E., Felip E., Holgado E. (2015). Nivolumab versus docetaxel in advanced nonsquamous non–small-cell lung cancer. New Engl. J. Med..

[54] Borghaei H., Gettinger S., Vokes E.E., Chow L.Q., Burgio M.A., de Castro Carpeno J., Pluzanski A., Arrieta O., Frontera O.A., Chiari R. (2021). Five-year outcomes from the randomized, phase III trials checkmate 017 and 057: Nivolumab versus docetaxel in previously treated non–small-cell lung cancer. J. Clin. Oncol..

[55] Lynch T.J., Bondarenko I., Luft A., Serwatowski P., Barlesi F., Chacko R., Sebastian M., Neal J., Lu H., Cuillerot J.M. (2012). Ipilimumab in combination with paclitaxel and carboplatin as first-line treatment in stage IIIB/IV non-small-cell lung cancer: Results from a randomized, double-blind, multicenter phase II study. J. Clin. Oncol..

[56] Callahan M.K., Kluger H., Postow M.A., Segal N.H., Lesokhin A., Atkins M.B., Kirkwood J.M., Krishnan S., Bhore R., Horak C. (2018). Nivolumab Plus Ipilimumab in Patients with Advanced Melanoma: Updated Survival, Response, and Safety Data in a Phase I Dose-Escalation Study. J. Clin. Oncol..

[57] Reck M., Ciuleanu T.E., Cobo M., Schenker M., Zurawski B., Menezes J., Richardet E., Bennouna J., Felip E., Juan-Vidal O. (2023). First-line nivolumab plus ipilimumab with two cycles of chemotherapy versus chemotherapy alone (four cycles) in metastatic non-small cell lung cancer: CheckMate 9LA 2-year patient-reported outcomes. Eur. J. Cancer.

[58] Gandhi L., Rodríguez-Abreu D., Gadgeel S., Esteban E., Felip E., De Angelis F., Domine M., Clingan P., Hochmair M.J., Powell S.F. (2018). Pembrolizumab plus chemotherapy in metastatic non–small-cell lung cancer. N. Engl. J. Med..

[59] Paz-Ares L., Luft A., Vicente D., Tafreshi A., Gümüş M., Mazières J., Hermes B., Çay Şenler F., Csőszi T., Fülöp A. (2018). Pembrolizumab plus chemotherapy for squamous non–small-cell lung cancer. N. Engl. J. Med..

[60] Socinski M.A., Jotte R.M., Cappuzzo F., Orlandi F., Stroyakovskiy D., Nogami N., Rodríguez-Abreu D., Moro-Sibilot D., Thomas C.A., Barlesi F. (2018). Atezolizumab for first-line treatment of metastatic nonsquamous NSCLC. N. Engl. J. Med..

[61] de Goeje P.L., Poncin M., Bezemer K., Kaijen-Lambers M.E.H., Groen H.J.M., Smit E.F., Dingemans A.C., Kunert A., Hendriks R.W., Aerts J. (2019). Induction of Peripheral Effector CD8 T-cell Proliferation by Combination of Paclitaxel, Carboplatin, and Bevacizumab in Non-small Cell Lung Cancer Patients. Clin. Cancer Res..

[62] Govindan R., Szczesna A., Ahn M.J., Schneider C.P., Gonzalez Mella P.F., Barlesi F., Han B., Ganea D.E., Von Pawel J., Vladimirov V. (2017). Phase III Trial of Ipilimumab Combined with Paclitaxel and Carboplatin in Advanced Squamous Non-Small-Cell Lung Cancer. J. Clin. Oncol..

[63] Formenti S.C., Rudqvist N.P., Golden E., Cooper B., Wennerberg E., Lhuillier C., Vanpouille-Box C., Friedman K., Ferrari de Andrade L., Wucherpfennig K.W. (2018). Radiotherapy induces responses of lung cancer to CTLA-4 blockade. Nat. Med..

[64] Chalmers A.W., Patel S., Boucher K., Cannon L., Esplin M., Luckart J., Graves N., Van Duren T., Akerley W. (2019). Phase I Trial of Targeted EGFR or ALK Therapy with Ipilimumab in Metastatic NSCLC with Long-Term Follow-Up. Target Oncol..

[65] Zucali P.A., Lin C.C., Carthon B.C., Bauer T.M., Tucci M., Italiano A., Iacovelli R., Su W.C., Massard C., Saleh M. (2022). Targeting CD38 and PD-1 with isatuximab plus cemiplimab in patients with advanced solid malignancies: Results from a phase I/II open-label, multicenter study. J. Immunother. Cancer.

[66] Hellmann M.D., Rizvi N.A., Goldman J.W., Gettinger S.N., Borghaei H., Brahmer J.R., Ready N.E., Gerber D.E., Chow L.Q., Juergens R.A. (2017). Nivolumab plus ipilimumab as first-line treatment for advanced non-small-cell lung cancer (CheckMate 012): Results of an open-label, phase 1, multicohort study. Lancet Oncol..

[67] Paz-Ares L., Ciuleanu T.E., Cobo M., Schenker M., Zurawski B., Menezes J., Richardet E., Bennouna J., Felip E., Juan-Vidal O. (2021). First-line nivolumab plus ipilimumab combined with two cycles of chemotherapy in patients with non-small-cell lung cancer (CheckMate 9LA): An international, randomised, open-label, phase 3 trial. Lancet Oncol..

[68] Clarke J.M., Patel J.D., Robert F., Kio E.A., Thara E., Camidge D.R., Dunbar M., Nuthalapati S., Dinh M.H., Bach B.A. (2021). Veliparib and nivolumab in combination with platinum doublet chemotherapy in patients with metastatic or advanced non-small cell lung cancer: A phase 1 dose escalation study. Lung Cancer.

[69] Rizvi N.A., Hellmann M.D., Brahmer J.R., Juergens R.A., Borghaei H., Gettinger S., Chow L.Q., Gerber D.E., Laurie S.A., Goldman J.W. (2016). Nivolumab in Combination with Platinum-Based Doublet Chemotherapy for First-Line Treatment of Advanced Non-Small-Cell Lung Cancer. J. Clin. Oncol..

[70] Ready N., Hellmann M.D., Awad M.M., Otterson G.A., Gutierrez M., Gainor J.F., Borghaei H., Jolivet J., Horn L., Mates M. (2019). First-Line Nivolumab Plus Ipilimumab in Advanced Non-Small-Cell Lung Cancer (CheckMate 568): Outcomes by Programmed Death Ligand 1 and Tumor Mutational Burden as Biomarkers. J. Clin. Oncol..

[71] Gettinger S.N., Redman M.W., Bazhenova L., Hirsch F.R., Mack P.C., Schwartz L.H., Bradley J.D., Stinchcombe T.E., Leighl N.B., Ramalingam S.S. (2021). Nivolumab Plus Ipilimumab vs. Nivolumab for Previously Treated Patients with Stage IV Squamous Cell Lung Cancer: The Lung-MAP S1400I Phase 3 Randomized Clinical Trial. JAMA Oncol..

[72] Rajan A., Gray J.E., Devarakonda S., Birhiray R., Korchin B., Menius E., Donahue R.N., Schlom J., Gulley J.L. (2023). Phase 1 trial of CV301 in combination with anti-PD-1 therapy in nonsquamous non-small cell lung cancer. Int. J. Cancer.

[73] Ott P.A., Hu-Lieskovan S., Chmielowski B., Govindan R., Naing A., Bhardwaj N., Margolin K., Awad M.M., Hellmann M.D., Lin J.J. (2020). A Phase Ib Trial of Personalized Neoantigen Therapy Plus Anti-PD-1 in Patients with Advanced Melanoma, Non-small Cell Lung Cancer, or Bladder Cancer. Cell.

[74] Leighl N.B., Redman M.W., Rizvi N., Hirsch F.R., Mack P.C., Schwartz L.H., Wade J.L., Irvin W.J., Reddy S.C., Crawford J. (2021). Phase II study of durvalumab plus tremelimumab as therapy for patients with previously treated anti-PD-1/PD-L1 resistant stage IV squamous cell lung cancer (Lung-MAP substudy S1400F, NCT03373760). J. Immunother. Cancer.

[75] Leighl N.B., Laurie S.A., Goss G.D., Hughes B.G.M., Stockler M., Tsao M.S., Hwang D.M., Joubert P., Kulkarni S., Blais N. (2022). CCTG BR34: A Randomized Phase 2 Trial of Durvalumab and Tremelimumab with or without Platinum-Based Chemotherapy in Patients with Metastatic NSCLC. J. Thorac. Oncol..

[76] Bang Y.J., Golan T., Dahan L., Fu S., Moreno V., Park K., Geva R., De Braud F., Wainberg Z.A., Reck M. (2020). Ramucirumab and durvalumab for previously treated, advanced non-small-cell lung cancer, gastric/gastro-oesophageal junction adenocarcinoma, or hepatocellular carcinoma: An open-label, phase Ia/b study (JVDJ). Eur. J. Cancer.

[77] Creelan B.C., Yeh T.C., Kim S.W., Nogami N., Kim D.W., Chow L.Q.M., Kanda S., Taylor R., Tang W., Tang M. (2021). A Phase 1 study of gefitinib combined with durvalumab in EGFR TKI-naive patients with EGFR mutation-positive locally advanced/metastatic non-small-cell lung cancer. Br. J. Cancer.

[78] Falchook G.S., Peeters M., Rottey S., Dirix L.Y., Obermannova R., Cohen J.E., Perets R., Frommer R.S., Bauer T.M., Wang J.S. (2021). A phase 1a/1b trial of CSF-1R inhibitor LY3022855 in combination with durvalumab or tremelimumab in patients with advanced solid tumors. Investig. New Drugs.

[79] Lim E.A., Bendell J.C., Falchook G.S., Bauer T.M., Drake C.G., Choe J.H., George D.J., Karlix J.L., Ulahannan S., Sachsenmeier K.F. (2022). Phase Ia/b, Open-Label, Multicenter Study of AZD4635 (an Adenosine A2A Receptor Antagonist) as Monotherapy or Combined with Durvalumab, in Patients with Solid Tumors. Clin. Cancer Res..

[80] Yang J.C., Shepherd F.A., Kim D.W., Lee G.W., Lee J.S., Chang G.C., Lee S.S., Wei Y.F., Lee Y.G., Laus G. (2019). Osimertinib Plus Durvalumab versus Osimertinib Monotherapy in EGFR T790M-Positive NSCLC following Previous EGFR TKI Therapy: CAURAL Brief Report. J. Thorac. Oncol..

[81] Jotte R., Cappuzzo F., Vynnychenko I., Stroyakovskiy D., Rodríguez-Abreu D., Hussein M., Soo R., Conter H.J., Kozuki T., Huang K.-C. (2020). Atezolizumab in Combination with Carboplatin and Nab-Paclitaxel in Advanced Squamous NSCLC (IMpower131): Results from a Randomized Phase III Trial. J. Thorac. Oncol..

[82] Vergnenegre A., Monnet I., Bizieux A., Bernardi M., Chiapa A.M., Léna H., Chouaïd C., Robinet G. (2020). Open-label Phase II trial to evaluate safety and efficacy of second-line metronomic oral vinorelbine-atezolizumab combination for stage-IV non-small-cell lung cancer—VinMetAtezo trial, (GFPC(‡) 04-2017). Future Oncol..

[83] West H., McCleod M., Hussein M., Morabito A., Rittmeyer A., Conter H.J., Kopp H.-G., Daniel D., McCune S., Mekhail T. (2019). Atezolizumab in combination with carboplatin plus nab-paclitaxel chemotherapy compared with chemotherapy alone as first-line treatment for metastatic non-squamous non-small-cell lung cancer (IMpower130): A multicentre, randomised, open-label, phase 3 trial. Lancet Oncol..

[84] Nishio M., Barlesi F., West H., Ball S., Bordoni R., Cobo M., Longeras P.D., Goldschmidt J., Novello S., Orlandi F. (2021). Atezolizumab Plus Chemotherapy for First-Line Treatment of Nonsquamous NSCLC: Results from the Randomized Phase 3 IMpower132 Trial. J. Thorac. Oncol..

[85] Blank C.U., Wong D.J., Ho T.H., Bauer T.M., Lee C.B., Bene-Tchaleu F., Zhu J., Zhang X., Cha E., Sznol M. (2021). Phase Ib Study of Atezolizumab Plus Interferon-α with or without Bevacizumab in Patients with Metastatic Renal Cell Carcinoma and Other Solid Tumors. Curr. Oncol..

[86] Jung K.H., LoRusso P., Burris H., Gordon M., Bang Y.J., Hellmann M.D., Cervantes A., Ochoa de Olza M., Marabelle A., Hodi F.S. (2019). Phase I Study of the Indoleamine 2,3-Dioxygenase 1 (IDO1) Inhibitor Navoximod (GDC-0919) Administered with PD-L1 Inhibitor (Atezolizumab) in Advanced Solid Tumors. Clin. Cancer Res..

[87] Langer C.J., Gadgeel S.M., Borghaei H., Papadimitrakopoulou V.A., Patnaik A., Powell S.F., Gentzler R.D., Martins R.G., Stevenson J.P., Jalal S.I. (2016). Carboplatin and pemetrexed with or without pembrolizumab for advanced, non-squamous non-small-cell lung cancer: A randomised, phase 2 cohort of the open-label KEYNOTE-021 study. Lancet Oncol..

[88] Kurata T., Nakagawa K., Satouchi M., Seto T., Sawada T., Han S., Homma M., Noguchi K., Nogami N. (2021). Phase 1 study of pembrolizumab plus chemotherapy as first-line treatment in Japanese patients with advanced NSCLC. Cancer Treat. Res. Commun..

[89] Gadgeel S., Rodríguez-Abreu D., Speranza G., Esteban E., Felip E., Dómine M., Hui R., Hochmair M.J., Clingan P., Powell S.F. (2020). Updated Analysis from KEYNOTE-189: Pembrolizumab or Placebo Plus Pemetrexed and Platinum for Previously Untreated Metastatic Nonsquamous Non-Small-Cell Lung Cancer. J. Clin. Oncol..

[90] Arrieta O., Barrón F., Ramírez-Tirado L.A., Zatarain-Barrón Z.L., Cardona A.F., Díaz-García D., Ramos M.Y., Mota-Vega B., Carmona A., Álvarez M.P.P. (2020). Efficacy and Safety of Pembrolizumab Plus Docetaxel vs Docetaxel Alone in Patients with Previously Treated Advanced Non-Small Cell Lung Cancer: The PROLUNG Phase 2 Randomized Clinical Trial. JAMA Oncol..

[91] Herbst R.S., Arkenau H.T., Santana-Davila R., Calvo E., Paz-Ares L., Cassier P.A., Bendell J., Penel N., Krebs M.G., Martin-Liberal J. (2019). Ramucirumab plus pembrolizumab in patients with previously treated advanced non-small-cell lung cancer, gastro-oesophageal cancer, or urothelial carcinomas (JVDF): A multicohort, non-randomised, open-label, phase 1a/b trial. Lancet Oncol..

[92] Reckamp K.L., Redman M.W., Dragnev K.H., Minichiello K., Villaruz L.C., Faller B., Al Baghdadi T., Hines S., Everhart L., Highleyman L. (2022). Phase II Randomized Study of Ramucirumab and Pembrolizumab Versus Standard of Care in Advanced Non-Small-Cell Lung Cancer Previously Treated with Immunotherapy-Lung-MAP S1800A. J. Clin. Oncol..

[93] Taylor M.H., Lee C.H., Makker V., Rasco D., Dutcus C.E., Wu J., Stepan D.E., Shumaker R.C., Motzer R.J. (2020). Phase IB/II Trial of Lenvatinib Plus Pembrolizumab in Patients with Advanced Renal Cell Carcinoma, Endometrial Cancer, and Other Selected Advanced Solid Tumors. J. Clin. Oncol..

[94] Kitano S., Fujiwara Y., Shimizu T., Iwasa S., Yonemori K., Kondo S., Shimomura A., Koyama T., Ebata T., Ikezawa H. (2022). A feasibility study of lenvatinib plus pembrolizumab in Japanese patients with advanced solid tumors. Cancer Chemother. Pharmacol..

[95] Naing A., Wong D.J., Infante J.R., Korn W.M., Aljumaily R., Papadopoulos K.P., Autio K.A., Pant S., Bauer T.M., Drakaki A. (2019). Pegilodecakin combined with pembrolizumab or nivolumab for patients with advanced solid tumours (IVY): A multicentre, multicohort, open-label, phase 1b trial. Lancet Oncol..

[96] Besse B., Lopez P.G., Puente J., Cortot A., Garcia M.E.O., Perol M., Gil M., Chao G., Shahidi J., Bennouna J. (2017). Efficacy and safety of necitumumab and pembrolizumab combination therapy in patients with stage IV non-small cell lung cancer (NSCLC). Ann. Oncol..

[97] Levy B.P., Giaccone G., Besse B., Felip E., Garassino M.C., Gomez M.D., Garrido P., Piperdi B., Ponce-Aix S., Menezes D. (2019). Randomised phase 2 study of pembrolizumab plus CC-486 versus pembrolizumab plus placebo in patients with previously treated advanced non-small cell lung cancer. Eur. J. Cancer.

[98] Levy B., Barlesi F., Paz-Ares L., Bennouna J., Erman M., Felip E., Isla D., Kim H.R., Kim S.W., Madelaine J. (2022). Phase II study of afatinib plus pembrolizumab in patients with squamous cell carcinoma of the lung following progression during or after first-line chemotherapy (LUX-Lung-IO). Lung Cancer.

[99] Park H., Shapiro G.I., Gao X., Mahipal A., Starr J., Furqan M., Singh P., Ahrorov A., Gandhi L., Ghosh A. (2022). Phase Ib study of eprenetapopt (APR-246) in combination with pembrolizumab in patients with advanced or metastatic solid tumors. ESMO Open.

[100] Ramalingam S.S., Thara E., Awad M.M., Dowlati A., Haque B., Stinchcombe T.E., Dy G.K., Spigel D.R., Lu S., Iyer Singh N. (2022). JASPER: Phase 2 trial of first-line niraparib plus pembrolizumab in patients with advanced non-small cell lung cancer. Cancer.

[101] Xiao A., Luke J.J., Partouche J., Karrison T., Chmura S.J., Al-Hallaq H.A. (2022). Evaluation of Dose Distribution to Organs-at-Risk in a Prospective Phase 1 Trial of Pembrolizumab and Multisite Stereotactic Body Radiation Therapy (SBRT). Pract. Radiat. Oncol..

[102] Felip E., Altorki N., Zhou C., Csőszi T., Vynnychenko I., Goloborodko O., Luft A., Akopov A., Martinez-Marti A., Kenmotsu H. (2021). Adjuvant atezolizumab after adjuvant chemotherapy in resected stage IB-IIIA non-small-cell lung cancer (IMpower010): A randomised, multicentre, open-label, phase 3 trial. Lancet.

[103] O’Brien M., Paz-Ares L., Marreaud S., Dafni U., Oselin K., Havel L., Esteban E., Isla D., Martinez-Marti A., Faehling M. (2022). Pembrolizumab versus placebo as adjuvant therapy for completely resected stage IB-IIIA non-small-cell lung cancer (PEARLS/KEYNOTE-091): An interim analysis of a randomised, triple-blind, phase 3 trial. Lancet Oncol..

[104] Forde P.M., Chaft J.E., Smith K.N., Anagnostou V., Cottrell T.R., Hellmann M.D., Zahurak M., Yang S.C., Jones D.R., Broderick S. (2018). Neoadjuvant PD-1 Blockade in Resectable Lung Cancer. N. Engl. J. Med..

[105] Cascone T., William W.N., Weissferdt A., Leung C.H., Lin H.Y., Pataer A., Godoy M.C.B., Carter B.W., Federico L., Reuben A. (2021). Neoadjuvant nivolumab or nivolumab plus ipilimumab in operable non-small cell lung cancer: The phase 2 randomized NEOSTAR trial. Nat. Med..

[106] Forde P.M., Spicer J., Lu S., Provencio M., Mitsudomi T., Awad M.M., Felip E., Broderick S.R., Brahmer J.R., Swanson S.J. (2022). Neoadjuvant Nivolumab plus Chemotherapy in Resectable Lung Cancer. N. Engl. J. Med..

[107] Provencio M., Nadal E., Insa A., García-Campelo M.R., Casal-Rubio J., Dómine M., Majem M., Rodríguez-Abreu D., Martínez-Martí A., De Castro Carpeño J. (2020). Neoadjuvant chemotherapy and nivolumab in resectable non-small-cell lung cancer (NADIM): An open-label, multicentre, single-arm, phase 2 trial. Lancet Oncol..

[108] Cascone T., Provencio M., Sepesi B., Lu S., Aanur N., Li S., Spicer J. (2020). Checkmate 77T: A phase III trial of neoadjuvant nivolumab (NIVO) plus chemotherapy (chemo) followed by adjuvant nivo in resectable early-stage NSCLC. J. Clin. Oncol..

[109] Eichhorn F., Klotz L.V., Bischoff H., Thomas M., Lasitschka F., Winter H., Hoffmann H., Eichhorn M.E. (2019). Neoadjuvant anti-programmed Death-1 immunotherapy by Pembrolizumab in resectable nodal positive stage II/IIIa non-small-cell lung cancer (NSCLC): The NEOMUN trial. BMC Cancer.

[110] Yi J.S., Ready N., Healy P., Dumbauld C., Osborne R., Berry M., Shoemaker D., Clarke J., Crawford J., Tong B. (2017). Immune Activation in Early-Stage Non-Small Cell Lung Cancer Patients Receiving Neoadjuvant Chemotherapy Plus Ipilimumab. Clin. Cancer Res..

[111] Rothschild S.I., Zippelius A., Eboulet E.I., Savic Prince S., Betticher D., Bettini A., Früh M., Joerger M., Lardinois D., Gelpke H. (2021). SAKK 16/14: Durvalumab in Addition to Neoadjuvant Chemotherapy in Patients with Stage IIIA(N2) Non-Small-Cell Lung Cancer-A Multicenter Single-Arm Phase II Trial. J. Clin. Oncol..

[112] Wislez M., Mazieres J., Lavole A., Zalcman G., Carre O., Egenod T., Caliandro R., Dubos-Arvis C., Jeannin G., Molinier O. (2022). Neoadjuvant durvalumab for resectable non-small-cell lung cancer (NSCLC): Results from a multicenter study (IFCT-1601 IONESCO). J. Immunother. Cancer.

[113] Heymach J.V., Mitsudomi T., Harpole D., Aperghis M., Jones S., Mann H., Fouad T.M., Reck M. (2022). Design and Rationale for a Phase III, Double-Blind, Placebo-Controlled Study of Neoadjuvant Durvalumab + Chemotherapy Followed by Adjuvant Durvalumab for the Treatment of Patients with Resectable Stages II and III non-small-cell Lung Cancer: The AEGEAN Trial. Clin. Lung Cancer.

[114] Campelo R.G., Forde P., Weder W., Spicer J., He P., Hamid O., Martinez P., Cascone T. (2019). P2. 04-28 NeoCOAST: Neoadjuvant Durvalumab Alone or with Novel Agents for Resectable, Early-Stage (I–IIIA) Non-Small Cell Lung Cancer. J. Thorac. Oncol..

[115] Chaft J.E., Oezkan F., Kris M.G., Bunn P.A., Wistuba I.I., Kwiatkowski D.J., Owen D.H., Tang Y., Johnson B.E., Lee J.M. (2022). Neoadjuvant atezolizumab for resectable non-small cell lung cancer: An open-label, single-arm phase II trial. Nat. Med..

[116] Forde P.M., Spicer J., Lu S., Provencio M., Mitsudomi T., Awad M.M., Felip E., Broderick S., Brahmer J., Swanson S.J. (2021). Abstract CT003: Nivolumab (NIVO)+ platinum-doublet chemotherapy (chemo) vs chemo as neoadjuvant treatment (tx) for resectable (IB-IIIA) non-small cell lung cancer (NSCLC) in the phase 3 CheckMate 816 trial. Cancer Res..

[117] Paz-Ares L., Hasan B., Dafni U., Menis J., De Maio E., Oselin K., Albert I., Faehling M., Van Schil P., O’Brien M.E.R. (2017). A randomized, phase 3 trial with anti-PD-1 monoclonal antibody pembrolizumab (MK-3475) versus placebo for patients with early stage NSCLC after resection and completion of standard adjuvant therapy (EORTC/ETOP 1416-PEARLS). Ann. Oncol..

[118] Naidoo J., Wang X., Woo K.M., Iyriboz T., Halpenny D., Cunningham J., Chaft J.E., Segal N.H., Callahan M.K., Lesokhin A.M. (2017). Pneumonitis in patients treated with anti–programmed death-1/programmed death ligand 1 therapy. J. Clin. Oncol..

[119] Conroy M., Naidoo J. (2022). Immune-related adverse events and the balancing act of immunotherapy. Nat. Commun..

[120] Hsu M.L., Murray J.C., Psoter K.J., Zhang J., Barasa D., Brahmer J.R., Ettinger D.S., Forde P.M., Hann C.L., Lam V.K. (2022). Clinical Features, Survival, and Burden of Toxicities in Survivors More Than One Year after Lung Cancer Immunotherapy. Oncologist.

[121] Reck M., Rodríguez-Abreu D., Robinson A.G., Hui R., Csőszi T., Fülöp A., Gottfried M., Peled N., Tafreshi A., Cuffe S. (2019). Updated Analysis of KEYNOTE-024: Pembrolizumab versus Platinum-Based Chemotherapy for Advanced Non-Small-Cell Lung Cancer with PD-L1 Tumor Proportion Score of 50% or Greater. J. Clin. Oncol..

[122] Johnson D.B., Balko J.M., Compton M.L., Chalkias S., Gorham J., Xu Y., Hicks M., Puzanov I., Alexander M.R., Bloomer T.L. (2016). Fulminant myocarditis with combination immune checkpoint blockade. N. Engl. J. Med..

[123] Suresh K., Naidoo J., Zhong Q., Xiong Y., Mammen J., De Flores M.V., Cappelli L., Balaji A., Palmer T., Forde P.M. (2019). The alveolar immune cell landscape is dysregulated in checkpoint inhibitor pneumonitis. J. Clin. Investig..

[124] Hailemichael Y., Johnson D.H., Abdel-Wahab N., Foo W.C., Bentebibel S.-E., Daher M., Haymaker C., Wani K., Saberian C., Ogata D. (2022). Interleukin-6 blockade abrogates immunotherapy toxicity and promotes tumor immunity. Cancer Cell.

[125] Weinmann S.C., Pisetsky D.S. (2019). Mechanisms of immune-related adverse events during the treatment of cancer with immune checkpoint inhibitors. Rheumatology.

[126] Haanen J., Obeid M., Spain L., Carbonnel F., Wang Y., Robert C., Lyon A.R., Wick W., Kostine M., Peters S. (2022). Management of toxicities from immunotherapy: ESMO Clinical Practice Guideline for diagnosis, treatment and follow-up^☆^. Ann. Oncol..

[127] Thompson J.A., Schneider B.J., Brahmer J., Achufusi A., Armand P., Berkenstock M.K., Bhatia S., Budde L.E., Chokshi S., Davies M. (2022). Management of Immunotherapy-Related Toxicities, Version 1.2022, NCCN Clinical Practice Guidelines in Oncology. J. Natl. Compr. Cancer Netw..

[128] Naidoo J., Zhang J., Lipson E.J., Forde P.M., Suresh K., Moseley K.F., Mehta S., Kwatra S.G., Parian A.M., Kim A.K. (2019). A Multidisciplinary Toxicity Team for Cancer Immunotherapy–Related Adverse Events. J. Natl. Compr. Cancer Netw..

[129] Cadranel J., Canellas A., Matton L., Darrason M., Parrot A., Naccache J.-M., Lavolé A., Ruppert A.-M., Fallet V. (2019). Pulmonary complications of immune checkpoint inhibitors in patients with nonsmall cell lung cancer. Eur. Respir. Rev..

[130] Garon E.B., Rizvi N.A., Hui R., Leighl N., Balmanoukian A.S., Eder J.P., Patnaik A., Aggarwal C., Gubens M., Horn L. (2015). Pembrolizumab for the treatment of non-small-cell lung cancer. N. Engl. J. Med..

[131] Hui R., Garon E.B., Goldman J.W., Leighl N.B., Hellmann M.D., Patnaik A., Gandhi L., Eder J.P., Ahn M.J., Horn L. (2017). Pembrolizumab as first-line therapy for patients with PD-L1-positive advanced non-small cell lung cancer: A phase 1 trial. Ann. Oncol..

[132] Yu H., Boyle T.A., Zhou C., Rimm D.L., Hirsch F.R. (2016). PD-L1 expression in lung cancer. J. Thorac. Oncol..

[133] Binnewies M., Roberts E.W., Kersten K., Chan V., Fearon D.F., Merad M., Coussens L.M., Gabrilovich D.I., Ostrand-Rosenberg S., Hedrick C.C. (2018). Understanding the tumor immune microenvironment (TIME) for effective therapy. Nat. Med..

[134] McGranahan N., Swanton C. (2015). Biological and therapeutic impact of intratumor heterogeneity in cancer evolution. Cancer Cell.

[135] Sha D., Jin Z., Budczies J., Kluck K., Stenzinger A., Sinicrope F.A. (2020). Tumor mutational burden as a predictive biomarker in solid tumors. Cancer Discov..

[136] Marabelle A., Fakih M., Lopez J., Shah M., Shapira-Frommer R., Nakagawa K., Chung H.C., Kindler H.L., Lopez-Martin J.A., Miller W.H. (2020). Association of tumour mutational burden with outcomes in patients with advanced solid tumours treated with pembrolizumab: Prospective biomarker analysis of the multicohort, open-label, phase 2 KEYNOTE-158 study. Lancet Oncol..

[137] Li H., van der Merwe P.A., Sivakumar S. (2022). Biomarkers of response to PD-1 pathway blockade. Br. J. Cancer.

[138] McGranahan N., Furness A.J., Rosenthal R., Ramskov S., Lyngaa R., Saini S.K., Jamal-Hanjani M., Wilson G.A., Birkbak N.J., Hiley C.T. (2016). Clonal neoantigens elicit T cell immunoreactivity and sensitivity to immune checkpoint blockade. Science.

[139] Wan J.C., Massie C., Garcia-Corbacho J., Mouliere F., Brenton J.D., Caldas C., Pacey S., Baird R., Rosenfeld N. (2017). Liquid biopsies come of age: Towards implementation of circulating tumour DNA. Nat. Rev. Cancer.

[140] Guibert N., Jones G., Beeler J.F., Plagnol V., Morris C., Mourlanette J., Delaunay M., Keller L., Rouquette I., Favre G. (2019). Targeted sequencing of plasma cell-free DNA to predict response to PD1 inhibitors in advanced non-small cell lung cancer. Lung Cancer.

[141] Goldberg S.B., Narayan A., Kole A.J., Decker R.H., Teysir J., Carriero N.J., Lee A., Nemati R., Nath S.K., Mane S.M. (2018). Early Assessment of Lung Cancer Immunotherapy Response via Circulating Tumor DNAAssessment of Immunotherapy Response by ctDNA. Clin. Cancer Res..

[142] Giroux Leprieur E., Herbretau G., Dumenil C., Julie C., Giraud V., Labrune S., Dumoulin J., Tisserand J., Emile J.-F., Blons H. (2018). Circulating tumor DNA evaluated by next-generation sequencing is predictive of tumor response and prolonged clinical benefit with nivolumab in advanced non-small cell lung cancer. Oncoimmunology.

[143] Zhang H., Nabel C.S., Li D., O’Connor R.Í., Crosby C.R., Chang S.M., Hao Y., Stanley R., Sahu S., Levin D.S. (2023). Histone Deacetylase 6 Inhibition Exploits Selective Metabolic Vulnerabilities in LKB1 Mutant, KRAS Driven NSCLC. J. Thorac. Oncol..

[144] Skoulidis F., Goldberg M.E., Greenawalt D.M., Hellmann M.D., Awad M.M., Gainor J.F., Schrock A.B., Hartmaier R.J., Trabucco S.E., Gay L. (2018). STK11/LKB1 Mutations and PD-1 Inhibitor Resistance in KRAS-Mutant Lung AdenocarcinomaSTK11/LKB1 Mutations and PD-1 Inhibitor Resistance in KRAS-Mutant LUAC. Cancer Discov..

[145] Boutsikou E., Domvri K., Hardavella G., Tsiouda D., Zarogoulidis K., Kontakiotis T. (2018). Tumour necrosis factor, interferon-gamma and interleukins as predictive markers of antiprogrammed cell-death protein-1 treatment in advanced non-small cell lung cancer: A pragmatic approach in clinical practice. Ther. Adv. Med. Oncol..

[146] Fan J., Yin Z., Xu J., Wu F., Huang Q., Yang L., Jin Y., Yang G. (2020). Circulating microRNAs predict the response to anti-PD-1 therapy in non-small cell lung cancer. Genomics.

[147] Li P., Qin P., Fu X., Zhang G., Yan X., Zhang M., Zhang X., Yang J., Wang H., Ma Z. (2021). Associations between peripheral blood lymphocyte subsets and clinical outcomes in patients with lung cancer treated with immune checkpoint inhibitor. Ann. Palliat. Med..

[148] Ferrara R., Naigeon M., Auclin E., Duchemann B., Cassard L., Jouniaux J.-M., Boselli L., Grivel J., Desnoyer A., Mezquita L. (2021). Circulating T-cell Immunosenescence in Patients with Advanced Non–small Cell Lung Cancer Treated with Single-agent PD-1/PD-L1 Inhibitors or Platinum-based Chemotherapy Immunosenescence in NSCLC. Clin. Cancer Res..

[149] Araujo B., de Lima V., Hansen M., Spanggaard I., Rohrberg K., Reker Hadrup S., Lassen U., Svane I.M. (2021). Immune cell profiling of peripheral blood as signature for response during checkpoint inhibition across cancer types. Front. Oncol..

[150] Sakurai T., De Velasco M.A., Sakai K., Nagai T., Nishiyama H., Hashimoto K., Uemura H., Kawakami H., Nakagawa K., Ogata H. (2022). Integrative analysis of gut microbiome and host transcriptomes reveals associations between treatment outcomes and immunotherapy-induced colitis. Mol. Oncol..

[151] Routy B., Le Chatelier E., Derosa L., Duong C.P., Alou M.T., Daillère R., Fluckiger A., Messaoudene M., Rauber C., Roberti M.P. (2018). Gut microbiome influences efficacy of PD-1–based immunotherapy against epithelial tumors. Science.

[152] Cappelli L.C., Bingham C.O., Forde P.M., Anagnostou V., Brahmer J., Lipson E.J., Mammen J., Schollenberger M., Shah A.A., Darrah E. (2022). Anti-RA33 antibodies are present in a subset of patients with immune checkpoint inhibitor-induced inflammatory arthritis. RMD Open.

[153] Groha S., Alaiwi S.A., Xu W., Naranbhai V., Nassar A.H., Bakouny Z., El Zarif T., Saliby R.M., Wan G., Rajeh A. (2022). Germline variants associated with toxicity to immune checkpoint blockade. Nat. Med..

[154] Altorki N., Borczuk A., Saxena A., Port J., Stiles B., Lee B., Sanfilippo N., Ko E., Scheff R., Pua B. (2019). P2.04-92 Neoadjuvant Durvalumab with or without Sub-Ablative Stereotactic Radiotherapy (SBRT) in Patients with Resectable NSCLC (NCT02904954). J. Thorac. Oncol..

[155] Spicer J., Cascone T., Kar G., Zheng Y., Blando J., Tan T., Cheng M., Mager R., Hamid O., Soo-Hoo Y. (2022). 929MO—Platform study of neoadjuvant durvalumab (D) alone or combined with novel agents in patients (pts) with resectable, early-stage non-small cell lung cancer (NSCLC): Pharmacodynamic correlates and circulating tumor DNA (ctDNA) dynamics in the NeoCOAST study. Ann. Oncol..

[156] Long L., Zhang X., Chen F., Pan Q., Phiphatwatchara P., Zeng Y., Chen H. (2018). The promising immune checkpoint LAG-3: From tumor microenvironment to cancer immunotherapy. Genes Cancer.

[157] Tawbi H.A., Schadendorf D., Lipson E.J., Ascierto P.A., Matamala L., Castillo Gutiérrez E., Rutkowski P., Gogas H.J., Lao C.D., De Menezes J.J. (2022). Relatlimab and Nivolumab versus Nivolumab in Untreated Advanced Melanoma. N. Engl. J. Med..

[158] Morgensztern D., Chaudhry A., Iannotti N., Acevedo A., Balaburski G., Balogh A., Peters S. (2021). 1359TiP RELATIVITY-104: First-line relatlimab (RELA) + nivolumab (NIVO) with chemotherapy vs nivo with chemotherapy in stage IV or recurrent non-small cell lung cancer (NSCLC): A phase II, randomized, double-blind study. Ann. Oncol..

[159] Alix-Panabières C., Pantel K. (2021). Liquid biopsy: From discovery to clinical implementation. Mol. Oncol..

